# Effect of Femtosecond Laser-Assisted Versus Conventional Clear Corneal Incisions on Endothelial Cell Density and Surgical Efficiency in Cataract Surgery

**DOI:** 10.3390/jcm15020626

**Published:** 2026-01-13

**Authors:** Nikola Bobot, Gabriele Thumann, Martina Kropp, Zeljka Cvejic, Valentin Pajic, Vesko Onov, Filip Slezak, Bojan Pajic

**Affiliations:** 1 Eye Clinic ORASIS, Swiss Eye Research Foundation, 5734 Reinach AG, Switzerlandfilip.slezak@orasis.ch (F.S.); 2 Division of Ophthalmology, Department of Clinical Neurosciences, Geneva University Hospitals, 1205 Geneva, Switzerlandmartina.kropp@unige.ch (M.K.); 3 Experimental Ophthalmology, University of Geneva, 1205 Geneva, Switzerland; 4 Department of Physics, Faculty of Sciences, University of Novi Sad, 21000 Novi Sad, Serbia; zeljka.cvejic@df.uns.ac.rs; 5 Faculty of Medicine, Semmelweis University, 1094 Budapest, Hungary; 6 Faculty of Medicine of the Military Medical Academy, University of Defense, 11000 Belgrade, Serbia

**Keywords:** cataract surgery, femtosecond laser-assisted cataract surgery (FLACS), clear corneal incision, endothelial cell density, phacoemulsification parameters, surgical efficiency, corneal thickness

## Abstract

**Objectives:** To investigate the efficacy of femtosecond laser-assisted cataract surgery (FLACS) with the FEMTO LDV Z8 laser system in comparison to manual conventional cataract surgery (CCS). **Background:** Preservation of corneal endothelial integrity remains one of the most critical determinants of long-term visual quality after cataract surgery. The introduction of low-energy femtosecond laser systems has raised expectations for safer and more efficient procedures, particularity in cases with denser cataracts. **Methods:** This is a prospective, randomized study. Here, 38 eyes in the FLACS group and 40 in the CCS group were included. The changes in central corneal thickness (CCT), endothelial cell density (ECD), and best-corrected visual acuity (BCVA) were analyzed. In addition, the effective phacoemulsification time (EPT), the total phacoemulsification time (PT), and the intraoperative time (IT) were evaluated as a function of cataract grade. The total follow-up period was six weeks. **Results:** On postoperative day 1, BCVA improved significantly faster in the FLACS group (0.91 ± 0.14) compared with the CCS group (0.70 ± 0.17; *p* < 0.05). FLACS also demonstrated a significantly shorter EPT (1.01 ± 0.91 s) than CCS (1.61 ± 1.70 s; *p* < 0.05). No significant differences were observed between groups regarding postoperative ECD or CCT at any time point. No intraoperative or postoperative complications occurred. **Conclusions:** Low-energy FLACS achieved comparable endothelial safety to CCS, while providing significantly faster early visual recovery and reduced ultrasound energy use. These finding support the clinical value of FLACS in patients with moderate to dense cataracts.

## 1. Introduction

Cataracts are a common age-related condition that affects millions of people worldwide each year. If left untreated, cataracts can lead to blindness and are among the leading causes of visual impairment in adults. The condition causes the lens of the eye to become opaque, resulting in hazy vision and a diminished ability to focus on objects. Although cataracts develop gradually, surgical intervention becomes necessary once visual function is significantly impaired [1].

Modern cataract surgery can be performed using either conventional manual techniques, which involve making corneal incisions with a handheld knife, or femtosecond laser-assisted cataract surgery (FLACS), where the clear corneal incisions, capsulotomy, and lens fragmentation are created with a femtosecond laser. In both approaches, the surgical procedure involves corneal incisions, anterior laser capsulotomy, or manual capsulorhexis, removal of the cataractous lens, and implantation of an intraocular lens (IOL) to restore vision [1].

A precise, safe, and reproducible capsulotomy is a prerequisite for successful cataract surgery and IOL implantation. Compared to manual capsulorhexis, femtosecond laser-assisted capsulotomies have been reported in some studies to demonstrate less variation in centration and size with reproducible, uniform circular and accurate diameters. Consequently, they may contribute to a more effective lens positioning with reduced probability of IOL tilt and decentration [2,3].

The femtosecond laser operates by generating a pulse of energy with duration in the femtosecond range, which is precisely delivered in a focused manner to the tissue, causing a process called photodisruption that transforms the tissue in the focal region into plasma [4]. This plasma then rapidly expands by creating micro-cavitation bubbles that separate the tissue. Repeated pulses across an area result in an incision [5].

One of the recent advances in femtosecond lasers is the use of low energy. Low-energy technology is a particularly gentle method: whereas high-pulse-energy lasers rely on mechanically induced disruption through cavitation bubble effects, low-energy systems achieve effective tissue separation via plasma-mediated tissue ablation, without the need for secondary mechanical tearing [6]. Therefore, low-energy pulses result in a very smooth surface without damaging adjacent tissue [3,6,7]. Enlarging the numerical aperture of laser-focusing optics and increasing the repetition frequency of laser pulses further decreases collateral damage while increasing precision [7].

The femtosecond laser, FEMTO LDV 8 (Ziemer Ophthalmic Systems AG, Port, Switzerland), used in this study that operates with low pulse energy (nJ) and a high repetition rate in the MHz range features these outstanding properties. Additionally, it is a compact and mobile system usable in corneal refractive, therapeutic, and cataract surgeries. In cataract procedures, it can perform capsulotomies, lens fragmentation, and various types of corneal incisions. Its innovative technology (low energy combined with high-frequency pulses) has been associated in previous studies with reduced cellular apoptosis, smooth and strong capsulotomies, sustained mydriasis maintained throughout surgery, and minimal inflammation [2,8,9,10,11,12]. However, other studies have found that the concentration of prostaglandin in the anterior chamber correlates with the energy applied. This can lead to miosis during the use of a higher-energy, conventional femtosecond laser due to increased prostaglandin levels [13]. Despite increasing clinical adoption, the impact of femtosecond laser technology on corneal endothelial cell behavior remains controversial [9,14,15,16,17]. Some studies have shown a reduction in effective phacoemulsification time (EPT) and ultrasound energy with femtosecond laser pretreatment, suggesting potential protection of endothelial cells through reduced mechanical and thermal stress [9,14,15]. However, other studies have reported no significant advantages of FLACS over conventional techniques, particularly regarding endothelial preservation [16,17]. These discrepancies may arise from differences in cataract grading, energy settings, and optical interface design [6,7,18]. Therefore, assessing endothelial cell density (ECD) and morphology parameters such as coefficient of variation (CV) and hexagonality (6A) in conjunction with phacoemulsification parameters provides a comprehensive evaluation of corneal safety and surgical efficiency [19].

### Study Rationale and Hypothesis

The purpose of this study was to compare the surgical efficiency and early postoperative outcomes of femtosecond laser-assisted cataract surgery (FLACS) using the low-energy FEMTO LDV Z8 system with those of conventional clear corneal incision cataract surgery (CCS). Given the variability in published evidence regarding the effect of FLACS on corneal endothelial function, we formulated a neutral working hypothesis that endothelial cell density (ECD) and postoperative corneal thickness would remain comparable between the two techniques, while differences in ultrasound energy parameters might reflect the influence of the cataract grade and fragmentation method rather than an intrinsic superiority of one technique over the other. The rationale of this study was, therefore, to assess whether low-energy FLACS is associated with differences in intraoperative parameters—such as effective phacoemulsification time (EPT), ultrasound time (UT), and total intraoperative time (IT)—while maintaining corneal endothelial safety. In addition, we aimed to explore the extent to which baseline cataract grade affects surgical efficiency and endothelial outcomes in both techniques, given that nucleus density is a known determinant of phacoemulsification energy requirements. Primary outcome measures included surgical efficiency parameters (EPT, UT, and IT). Secondary outcome measures included best-corrected visual acuity (BCVA), endothelial cell density (ECD), central corneal thickness (CCT) pre- and post-operatively, effective phacoemulsification time (EPT), phacoemulsification time (PT), and intraoperative time (IT).

## 2. Materials and Methods

### 2.1. Study Design

This prospective, randomized study included 78 patients and 78 eyes. In patients who underwent surgery on both eyes, the side was randomized. The study was conducted at the ORASIS Eye Clinic in Reinach AG, Switzerland, from February 2020 to January 2024. The Cantonal Ethics Committee of Northwestern and Central Switzerland in Basel (BASEC ID: 2019-01166) granted authorization to conduct the study. The study was registered under ClinicalTrials.gov with the registration number NCT04082273. All patients were informed in detail and signed a consent form before the procedure, and the study was conducted in accordance with the principles of the Declaration of Helsinki. Randomization was performed using a simple 1:1 allocation sequence, assigning eyes either to the FLACS group or the CCS group. The resulting groups differed in baseline cataract grade, which is acknowledged as a methodological limitation and addressed in the Discussion Section. Eligible patients were those aged 40 years or older with visually significant cataracts requiring phacoemulsification and intraocular lens implantation, who were able to cooperate with the laser docking system, and who agreed to attend scheduled follow-up visits. Exclusion criteria included prior ocular surgery, corneal endothelial loss, uveitis, significant zonular weakness, and any condition that could compromise postoperative evolution of endothelial cell density or corneal thickness. The postoperative follow-up period was 6 weeks, allowing evaluation of early surgical outcomes but not long-term endothelial recovery.

### 2.2. Sample Size Justification

The sample size was determined by the number of eligible patients recruited during the study period. No formal power calculation was performed prior to enrollment, and the sample size, therefore, reflects the available population rather than a predefined statistical requirement.

### 2.3. Inclusion and Exclusion Criteria

Inclusion criteria were (1) age ≥ 40 years, (2) presence of senile cataract suitable for phacoemulsification with primary intraocular lens implantation, (3) ability to cooperate with the laser docking procedure, and (4) willingness to attend all postoperative visits. Exclusion criteria included glaucoma, pseudoexfoliation syndrome, corneal dystrophies or scars, previous surgery, active ocular inflammation, small pupils (<4 mm), or any condition likely to affect endothelial cell morphology or interfere with postoperative measurements.

All patients underwent a comprehensive preoperative examination. Cataract severity was graded using the Lens Opacities Classification System III (LOCS III).

Study subjects were randomly assigned in a 1:1 ratio to undergo either femtosecond laser-assisted cataract surgery (FLACS) or conventional cataract surgery (CCS), with one study eye per participant. When both eyes were eligible, the eye selected for surgery was determined by random allocation. All procedures were performed by the same experienced surgeon (B.P.).

For the FLACS group (38 eyes), who underwent clear corneal incisions, capsulotomy and lens fragmentation were performed using the (FEMTO LDV Z8) platform (Ziemer Ophthalmic System AG, Port, Switzerland), followed by ultrasound phacoemulsification using the Oertli CATARHEX3 (Oertli Instruments AG, Berneck, Switzerland) and IOL implantation.

For the CCS group (40 eyes), clear corneal incisions and capsulorhexis were created manually and the lens fragmentation was performed using the same phaco platform and surgical setting as in the FLACS group. In both groups, two paracenteses were routinely created to permit instrument maneuverability and maintain anterior chamber stability.

A monofocal aspheric intraocular lens (KOWA 2.2R) was implanted in the capsular bag in all patients.

Although randomization was applied, the baseline cataract grade differed between groups, with the FLACS group exhibiting a higher mean LOCS III nuclear opalescence score (from grade 0 to 5). This imbalance is acknowledged as a methodological limitation due to its potential influence on ultrasound energy requirements and endothelial outcomes.

### 2.4. Surgical Technique

Preoperative pupil dilation was performed using phenylephrine hydrochloride (5.4 mg) and tropicamide (0.28 mg; Mydriasert; Laboratoires Théa, Clermont-Ferrand, France). Mydriasert is available in tablet form and is inserted into the inferior fornix. Lidocaine 1% was administered intracamerally as a local anesthetic.

The FLACS group was used to perform capsulotomy, lens fragmentation, and corneal incisions. The eye was fixed with a suction ring at a target vacuum of 400 mbar. A balanced salt solution (BSS) was placed in the liquid interface before docking the laser handpiece onto the suction ring. The laser treatment sequence included the following:

- anterior capsulotomy with a targeted diameter of 5.0 mm,
- lens fragmentation into six radial segments combined with a concentric circular cut,
- creation of a 2.2 mm main incision and two 0.8 mm paracenteses.

Following laser pretreatment, standard ultrasound was performed to remove the pre-cut lens fragments. Cortical material was removed using an irrigation–aspiration system, and the capsular bag was polished when required. A monofocal aspheric intraocular lens was implanted into the capsular bag. Two 0.8 mm paracenteses were used in both FLACS and CCS procedures to allow safe bimanual instrument access and stable anterior chamber manipulation. This reflects the standardized surgical workflow at our center and is not related to the degree of lens nucleus pre-fragmentation.

CCS group: In the conventional cataract surgery group, two 0.8 mm side port incisions were first created with a steel lance. A manual continuous curvilinear capsulorhexis was formed with a cystotome. The target diameter was 5 mm. The main 2.2 mm clear corneal incision was subsequently created with the same instrument. After hydrodissection, the phaco chop technique was used to remove the cataract. The cortex remnants were removed by the irrigation–aspiration system, followed by IOL implantation in the capsular bag. The main incision was made at 145° on the right eye and 90° on the left. The side ports were positioned at 80° and 180° on the right and 110° and 0° on the left. The design of the access points was independent of the surgical method and identical in both groups.

Phacoemulsification platform: All ultrasound procedures were performed using the Oertli Catharex 3 (Oertli Instrumente AG, Berneck, Switzerland). Identical phaco settings were used in both groups to ensure procedural consistency (vacuum 600 mmHg and aspiration flow rate 50 mL/min). Effective phacoemulsification time (EPT) and ultrasound time (UT) were recorded directly from the device.

### 2.5. Performance, Safety, and Surgery-Related Outcomes

This study compared selected performance, safety, and surgery-related outcome variables between the FLACS group and CCS group. The variables included changes in CCT, ECD, and BCVA, and intraoperative ultrasound metrics recorded during phacoemulsification. Any intraoperative or postoperative complications during the follow-up period were recorded.

The preoperative assessment was conducted within four weeks prior to surgery, and the total follow-up duration was six weeks.

CCT was measured preoperatively and postoperatively at 1 day, 12 days, 4 weeks, and 6 weeks using the Galilei G2 system (Ziemer Ophthalmic Systems AG, Switzerland). ECD was assessed preoperatively and postoperatively at 12 days, 4 weeks, and 6 weeks using the EM-3000 endothelial microscope (Tomey Corporation, 2-11-33 Noritakeshinmachi, Nishi-Ku, Nagoya, Aichi 451-0051, Japan). Intraoperative ultrasound parameters were obtained from the phacoemulsification platform. This system reports effective phacoemulsification time (EPT) and ultrasound time (UT), which serve as validated indicators of intraoperative energy delivery for this device. The platform does not provide cumulative dissipated energy (CDE), aspiration time, or total fluid usage, which is acknowledged as a methodological limitation. Intraoperative time (IT) was defined as the interval between lid retractor insertion and removal. Laser docking and laser treatment time were not included in this interval, consistent with the methodology used in prior low-energy FLACS studies.

BCVA was evaluated preoperatively and at postoperative days 1 and 12, and at weeks 4 and 6, using a standardized Early Treatment Diabetic Retinopathy Study (ETDRS) chart.

### 2.6. Statistical Analysis

Statistical analyses were performed using SPSS version 22 (IBM Corp., Armonk, NY, USA). All statistical tests were two-tailed, and a *p*-value less than 0.05 was considered statistically significant. Continuous variables were inspected for outliers using boxplots, and the distribution of data was assessed for normality using both the Shapiro–Wilk and Kolmogorov–Smirnov tests. Depending on the distribution of data, either parametric or non-parametric tests were applied. For normally distributed variables, comparisons between groups were performed using the independent-samples *t*-test, whereas within-group changes over time were assessed using the paired *t*-test. For non-normally distributed variables, the Wilcoxon signed-rank test and Mann–Whitney U test were used. The descriptive statistics of continuous variables included the mean, standard deviation, median, minimum, and maximum values. To account for the potential influence of cataract grade on surgical outcomes, subgroup analyses were performed using one-way ANOVA with Tukey post hoc adjustments. These analyses were exploratory in nature, as the study was not powered to detect differences between cataract grade subgroups. Correlations between endothelial cell density (ECD) change and surgical parameters (EPT, UT, and IT) were examined using Pearson’s correlation coefficient. Visual acuity was converted into logMAR for statistical analysis in accordance with international reporting standards. Decimal notation is presented in figures solely for clinical interpretability.

## 3. Results

A total of 78 patients (45 male and 33 female) were enrolled. The FLACS group included 38 patients (20 male and 18 female), and the CCS group included 40 patients (25 male and 15 female). The mean age did not differ significantly between groups (FLACS: 69.5 ± 10.2; CCS: 69.4 ± 10.6).

**Figure 1:**
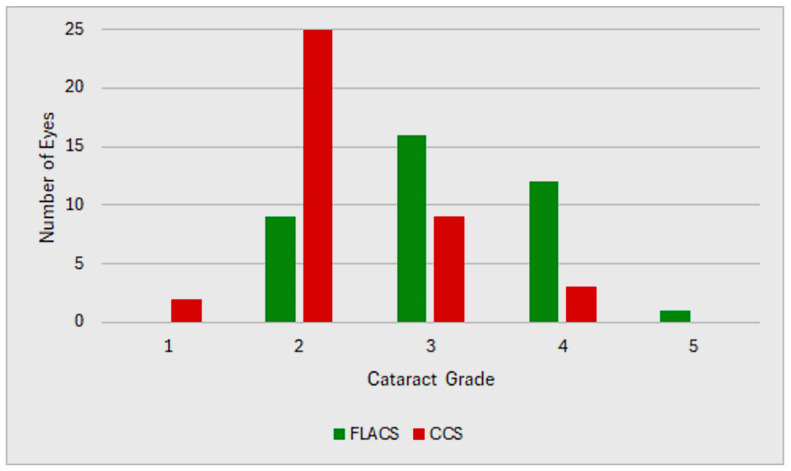
Distribution of cataract grading between the FLACS and CCS groups.

Unilateral cataract surgery was performed on 37 right and 41 left eyes. The mean cataract grade was significantly higher in the FLACS group (3.13 ± 0.81) compared with the CCS group (2.33 ± 0.68; *p* < 0.05), indicating a baseline imbalance between the groups (Figure 1). No intraoperative or postoperative complications occurred. All outcomes represent early postoperative changes within the six-week follow-up period.

### 3.1. Corneal Parameter Analysis

#### 3.1.1. Endothelial Corneal Cell Density (ECD)

ECD decreased over time in both FLACS and CCS groups. Between-group differences were not statistically significant at any time point (Table 1). Within-group reductions from baseline were significant at week 4 and week 6 in both groups. Percentage of endothelial cell loss at six weeks was 8.9% in the FLACS group and 7.4% in the CCS group—this difference was not statistically significant. Percentage values for all time points are provided in Supplementary Table S1.

#### 3.1.2. Grade-Stratified Analyses

Because subgroup sizes—particularly Grade 4 in the CCS arm—were small, all stratified analyses were exploratory and not powered for definitive comparations. Numerical differences were observed in some strata; however, these should be interpreted with caution. Apparent between-group differences in Grades 2, 2 + 3, and Grade 4 do not permit firm conclusions due to the limited subgroup sample sizes (Table 1).

#### 3.1.3. The Coefficient of Variation (CV) of Endothelial Cell Area

Mean CV values showed minimal changes over time in both groups, with no statistically significant between-group differences in the overall cohort or in Grades 2, 3, and 2 + 3 (Table 2). In Grade 4, numerical differences were observed at week 4 and week 6; however, these findings are exploratory owing to the small subgroup sizes.

#### 3.1.4. The Percentage of Hexagonal Cells (6A)

Mean 6A (%) decreased slightly after surgery in both groups, with no significant between-group differences for Grades 2, 3, or 2 + 3 (Table 3). In Grade 4, differences were detected from day 12 onward; however, these comparisons remain exploratory due to the very small number of Grade 4 CCS cases.

### 3.2. Central Corneal Thickness (CCT)

**Figure 2:**
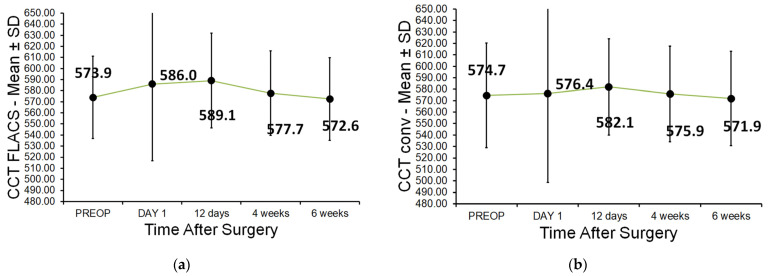
(**a**,**b**) Central corneal thickness (µm): (**a**) FLACS and (**b**) CCS.

No significant differences were observed between groups pre- or post-operatively at any time (day 1, day 12, week 4, or week 6). Mean ± SD CCT (µm) in FLACS vs. CCS, respectively: preoperative, 573.9 ± 37.1 vs. 574.7 ± 45.6; day 1, 586.0 ± 69.1 vs. 576.4 ± 77.7; day 12, 589.1 ± 42.8 vs. 582.1 ± 42.0; week 4, 577.7 ± 38.2 vs. 575.9 ± 41.9; week 6, 572.6 ± 37.3 vs. 571.9 ± 41.3 (Figure 2a,b).

### 3.3. Best-Corrected Visual Acuity (BCVA)

**Figure 3:**
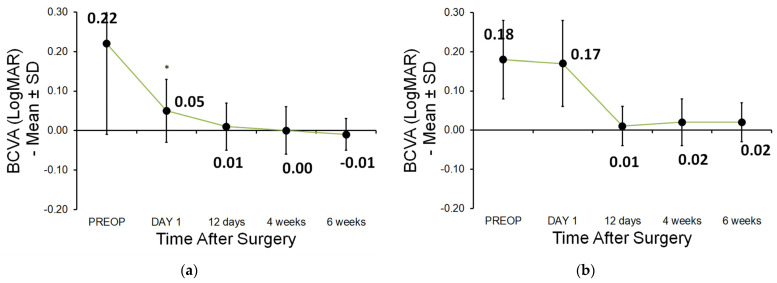
(**a**,**b**) Best-corrected visual acuity (BCVA; logMAR): (**a**) FLACS and (**b**) CCS (* *p* < 0.05 is considered statistically significant).

Statistical analysis of BCVA was performed using logMAR values. For clinical interpretation, decimal notation is shown in Figure 3. BCVA improved significantly faster in the FLACS group at day 1 (*p* < 0.001). At all subsequent visits (day 12, week 4, and week 6), there were no statistically significant differences in BCVA between FLACS and CCS (all *p* ≥ 0.05; Figure 3a,b).

### 3.4. Effective Phacoemulsification Time (EPT) and Ultrasound Time (UT)

Mean intraoperative EPT was significantly shorter in FLACS (1.01 ± 0.91 s) than in CCS (1.61 ± 1.70 s; *p* < 0.05). Ultrasound time (UT) was numerically shorter in FLACS (1.42 ± 1.23 s vs. 1.81 ± 1.63 s), but the difference did not reach statistical significance (*p* = 0.142). Grade-stratified analyses (≤2, =3, and ≥4) were performed as an exploratory comparision and should be interpreted with caution because subgroup sizes—especially in Grade ≥ 4 for CCS—were small:

- IT: Mean intraoperative time was shorter in FLACS than CCS in Grades ≤ 2 and =3 (*p* < 0.001 and *p* = 0.007, respectively). In Grade ≥ 4, the difference was not significant (*p* = 0.207; CCS, *n* = 3).
- EPT: For Grade ≤ 2, EPT was significantly shorter in FLACS (*p* = 0.046). Two-way ANOVA showed independent effects of cataract grade (*p* = 0.031) and surgical technique (*p* = 0.007) on EPT.
- UT: For Grade ≤ 2, UT was significantly shorter in FLACS (*p* = 0.030). Two-way ANOVA again showed independent effects of cataract grade (*p* = 0.003) and technique (*p* = 0.012) on UT.

### 3.5. Surgery Time (IT)

Intraoperative time (IT), defined as the interval from lid retractor insertion to removal, was significantly shorter with FLACS (6.97 ± 1.31 min) than CCS (9.90 ± 2.46 min; *p* < 0.05), indicating higher overall surgical efficiency. Pearson correlation analysis between changes in ECD and intraoperative parameters (EPT, UT, and IT) showed only weak, nonsignificant associations, suggesting that phacoemulsification metrics accounted for only a limited proportion of the variability in endothelial cell loss (Table 4).

## 4. Discussion

This study compared early postoperative corneal endothelial outcomes following low-energy femtosecond laser-assisted cataract surgery (FLACS) and conventional cataract surgery (CCS). Despite the significantly higher baseline grade in the FLACS group, no statistically significant differences in endothelial cell density (ECD) or central corneal thickness (CCT) were observed between groups at any postoperative time point. These findings indicate comparable endothelial responses between low-energy FLACS and CCS during the early healing phase. Morphometric parameters, including the coefficient of variation (CV) and the percentage of hexagonal cells (6A), remained stable in both groups across all visits. Minimal morphometric change is consistent with prior studies demonstrating that low-energy femtosecond systems induce limited collateral compared with high-energy laser platforms [6,7,17,18]. Because the present study was not powered to detect subtle morphologic differences, these results should be interpreted with caution.

Analysis of ultrasound energy metrics showed that FLACS was associated with significantly shorter effective phacoemulsification time (EPT) and a nonsignificant trend toward lower ultrasound time (UT). These findings align with previous research demonstrating that femtosecond laser pre-fragmentation reduces intraoperative ultrasound requirements [14,15,20,21,22]. The weak correlation between EPT/UT and endothelial cell loss observed here further supports the multifactorial nature of endothelial injury, as reported in early studies emphasizing the influence of nucleus density, fluidics, and anterior chamber stability [23,24,25,26].

Our results are consistent with prior reports showing no significant difference in endothelial cell loss between FLACV and conventional phacoemulsification [15,16,17,18,20]. Importantly, differences between femtosecond platforms must be considered when interpreting endothelial outcomes. High-pulse energy systems (microjoule level) generate large cavitation bubbles and greater mechanical stress and have been associated with endothelial changes equal to or greater than those observed after CCS [9,14,16]. In contrast, the low-energy high-frequency FEMTO LDV Z8 system used in this study operates with nanojoule-level pulses and minimal bubble expansion [6,7], which may contribute to the comparable endothelial outcomes observed here. Thus, the present findings apply specifically to low-energy systems and should not be generalized to high-energy femtosecond platforms.

CCT followed a similar trajectory in both groups, with a slight decrease toward the baseline by week 6. Prior studies comparing low-energy FLACS with CCS have also shown no significant postoperative differences in CCT [15,17]. Because CCT can fluctuate due to hydration, epithelial healing, and device variability, early postoperative measurements should not be interpreted as indicators of endothelial pump function.

Visual outcomes showed faster improvement in BCVA in the FLACS group on postoperative day 1, consistent with studies reporting rapid early visual recovery following laser-assisted capsulotomy and lens fragmentation [2,26,27,28,29,30,31,32]. From day 12 onward, BCVA was comparable between groups, aligning with earlier work demonstrating similar long-term visual outcomes after FLACS and CCS [20,24,32,33,34]. FLACS lens pre-fragmentation reduces mechanical stress and ultrasound exposure within the anterior chamber. Even if ECD and CCT values remain similar, reduced intraocular turbulence and minimized manipulation result in less temporary corneal edema and a clearer optical pathway in the early postoperative phase, potentially resulting in better early BCVA compared to conventional surgery [2,35]. Furthermore, phacoemulsification energy can reduce postoperative inflammation. This dynamic has already been described in other studies. This leads to clearer media and better early visual acuity despite similar structural corneal parameters [36].

### Limitations

This study has several limitations. First, the sample size was modest, particularly within cataract grade subgroups, which reduces statistical power and limits subgroup interpretation. Second, the imbalance in baseline cataract grade between FLACS and CCS introduces potential confounding that cannot be fully adjusted for. Third, the six-week follow-up period captures only early postoperative corneal responses and does not allow conclusions regarding long-term endothelial stability. Fourth, detailed intraoperative energy metrics, such as cumulative dissipated energy (CDE), aspiration time, and irrigation volume, were unavailable, as the phacoemulsification platform records only EPT and UT. Finally, morphometric parameters (CV and 6A) were not analyzed using longitudinal modeling, and the study was not powered to detect subtle morphometric differences.

## 5. Conclusions

Femtosecond laser-assisted cataract surgery (FLACS) using a low-energy, high-frequency platform demonstrated surgical efficiency and corneal safety compared to conventional cataract surgery (CCS), despite a higher baseline cataract grade in the FLACS group. Endothelial cell density, central corneal thickness, and endothelial morphology (CV and 6A) remained similar between techniques at all postoperative time points.

FLACS achieved a consistently shorter effective phacoemulsification time and reduced intraoperative time, contributing to faster early visual recovery. Although correlations between EPT/UT and endothelial loss were weak, the reduced intraocular energy supports the procedural advantages of laser fragmentation.

These results do not indicate endothelial superiority of FLACS over CCS. Rather, they confirm that low-energy FLACS maintains endothelial integrity while providing workflow and early visual benefits.

Larger, cataract-grade-stratified randomized trials with an extended follow-up are needed to further evaluate long-term corneal stability and the clinical relevance of reduced ultrasound energy exposure.

## Figures and Tables

**Table 1:** Endothelial cell density (cells/mm^2^) over time in FLACS vs. CCS (all eyes and by grade). * *p* < 0.05 is considered statistically significant.

	FLACS	CCS	*p*-Value
**All Grades**			
Preoperative	2428 ± 353	2434 ± 324	*p* = 0.856
Day 1	2323 ± 380	2326 ± 380	*p* = 0.776
Day 12	2322 ± 384	2311 ± 400	*p* = 0.280
Week 4	2295 ± 387	2261 ± 417	*p* = 0.432
Week 6	2213 ± 397	2254 ± 417	*p* = 0.225
**Grade 2**			
Preoperative	2508 ± 435	2421 ± 249	*p* = 0.906
Day 1	2445 ± 477	2290 ± 384	*p* = 0.398
Day 12	2452 ± 531	2200 ± 519	*p* = 0.048 *
Week 4	2314 ± 503	2147 ± 512	*p* = 0.767
Week 6	2440 ± 505	2146 ± 481	*p* = 0.040 *
**Grade 3**			
Preoperative	2300 ± 259	2423 ± 482	*p* = 0.260
Day 1	2244 ± 380	2343 ± 433	*p* = 0.249
Day 12	2280 ± 251	2282 ± 544	*p* = 0.726
Week 4	2251 ± 290	2263 ± 495	*p* = 0.779
Week 6	2241 ± 338	2235 ± 518	*p* = 0.441
**Grades 2 and 3**			
Preoperative	2438 ± 328	2402 ± 307	*p* = 0.476
Day 1	2314 ± 417	2275 ± 417	*p* = 0.390
Day 12	2340 ± 370	2188 ± 415	*p* = 0.021 *
Week 4	2274 ± 374	2146 ± 498	*p* = 0.024 *
Week 6	2307 ± 402	2140 ± 478	*p* = 0.024 *
**Grade 4**			
Preoperative	2447 ± 251	2285 ± 443	*p* = 0.593
Day 1	2212 ± 385	2287 ± 374	*p* = 0.655
Day 12	2085 ± 523	1624 ± 573	*p* = 0.010 *
Week 4	2084 ± 621	1869 ± 704	*p* = 0.011 *
Week 6	2247 ± 527	1956 ± 750	*p* = 0.011 *

**Table 2:** CV (%) of corneal endothelial cell area over time: FLACS vs. CCS, overall and stratified by cataract grade. * *p* < 0.05 is considered statistically significant.

	FLACS	CCS	*p*-Value
**All Grades**			
Preoperative	38.74 ± 8.04	37.51 ± 4.55	*p* = 0.539
Day 1	40.88 ± 7.12	41.35 ± 6.84	*p* = 0.933
Day 12	38.64 ± 5.48	39.10 ± 4.93	*p* = 0.410
Week 4	39.42 ± 4.90	39.50 ± 5.13	*p* = 0.933
Week 6	40.03 ± 7.00	40.30 ± 5.79	*p* = 0.362
**Grade 2**			
Preoperative	39.42 ± 12.50	37.44 ± 4.86	*p* = 0.833
Day 1	39.18 ± 4.26	42.59 ± 7.74	*p* = 0.293
Day 12	36.82 ± 4.85	39.28 ± 5.10	*p* = 0.888
Week 4	39.08 ± 5.89	39.76 ± 5.64	*p* = 0.833
Week 6	37.27 ± 4.92	40.40 ± 6.11	*p* = 0.018 *
**Grade 3**			
Pre-operative	39.94 ± 6.61	37.25 ± 2.32	*p* = 0.246
Preoperative	39.87 ± 5.51	38.86 ± 3.76	*p* = 0.686
Day 1	39.07 ± 4.98	38.56 ± 3.61	*p* = 0.726
Day 12	40.13 ± 5.08	38.44 ± 3.75	*p* = 0.445
Week 4	41.00 ± 5.59	40.00 ± 6.14	*p* = 0.314
**Grades 2 and 3**			
Preoperative	39.40 ± 6.28	37.39 ± 4.34	*p* = 0.454
Day 1	39.83 ± 5.21	41.69 ± 7.11	*p* = 0.329
Day 12	38.48 ± 5.08	39.09 ± 4.71	*p* = 0.276
Week 4	40.04 ± 5.47	39.41 ± 5.19	*p* = 0.267
Week 6	40.21 ± 5.52	40.29 ± 6.03	*p* = 0.532
**Grade 4**			
Preoperative	37.33 ± 5.14	40.33 ± 8.33	*p* = 0.285
Day 1	44.13 ± 5.10	43.50 ± 6.36	*p* = 0.157
Day 12	37.22 ± 3.67	41.67 ± 6.74	*p* = 0.285
Week 4	38.18 ± 3.55	43.67 ± 5.03	*p* = 0.019 *
Week 6	39.56 ± 3.29	44.00 ± 3.46	*p* = 0.017 *

**Table 3:** Percentage of hexagonal cells (6A, %) over time in FLACS vs. CCS (all eyes and by grade). * *p* < 0.05 is considered statistically significant.

	FLACS	CCS	*p*-Value
**All Grades**			
Preoperative	46.24 ± 7.49	45.23 ± 5.92	*p* = 0.491
Day 1	42.91 ± 6.35	42.09 ± 8.39	*p* = 0.923
Day 12	43.12 ± 6.05	42.30 ± 8.43	*p* = 0.604
Week 4	41.83 ± 7.87	42.15 ± 7.17	*p* = 0.818
Week 6	42.85 ± 7.95	41.68 ± 7.40	*p* = 0.173
**Grade 2**			
Preoperative	43.67 ± 8.69	45.08 ± 6.34	*p* = 0.888
Day 1	44.00 ± 5.48	41.41 ± 7.85	*p* = 0.237
Day 12	46.13 ± 6.73	42.44 ± 7.71	*p* = 0.528
Week 4	42.11 ± 7.04	41.96 ± 7.59	*p* = 0.953
Week 6	43.88 ± 7.30	41.76 ± 7.06	*p* = 0.726
**Grade 3**			
Preoperative	46.50 ± 7.38	45.50 ± 4.81	*p* = 0.833
Day 1	42.47 ± 8.00	42.29 ± 8.16	*p* = 0.916
Day 12	42.00 ± 6.60	43.22 ± 4.47	*p* = 0.624
Week 4	43.60 ± 6.13	42.56 ± 6.02	*p* = 0.779
Week 6	43.00 ± 6.84	40.33 ± 8.31	*p* = 0.574
**Grades 2 and 3**			
Preoperative	45.48 ± 7.82	45.18 ± 5.94	*p* = 0.831
Day 1	43.00 ± 7.12	41.62 ± 8.55	*p* = 0.322
Day 12	43.43 ± 6.80	42.65 ± 6.95	*p* = 0.300
Week 4	43.04 ± 7.62	42.12 ± 7.13	*p* = 0.345
Week 6	43.29 ± 7.21	41.38 ± 7.30	*p* = 0.391
**Grade 4**			
Preoperative	45.83 ± 5.73	42.67 ± 7.23	*p* = 0.285
Day 1	42.88 ± 4.29	38.00 ± 4.24	*p* = 0.180
Day 12	42.89 ± 3.95	29.33 ± 4.22	*p* = 0.011 *
Week 4	39.36 ± 4.52	32.00 ± 2.64	*p* = 0.012 *
Week 6	41.67 ± 5.27	31.00 ± 2.65	*p* = 0.011 *

**Table 4:** Pearson correlation coefficients between ECD change and intraoperative parameters.

Parameter	FLACS (r)	*p*-Value	CCS (r)	*p*-Value	Interpretation
EPT (s)	−0.312	0.032 *	−0.118	0.523	Weak negative correlation in FLACS; shorter EPT associated with less ECD loss
UT (s)	−0.204	0.118	−0.095	0.611	No statistically significant correlation in either group
IT (min)	−0.162	0.214	−0.084	0.654	No significant relationship between procedure duration and ECD change

* *p* < 0.05 is considered statistically significant.

## Data Availability

Data supporting the findings of this study are available upon reasonable request from the first and corresponding authors. The datasets are archived at the clinics where the patients were treated. Data are not publicly available due to privacy concerns.

## Supplementary Materials

The following supporting information can be downloaded at: https://www.mdpi.com/article/10.3390/jcm15020626/s1, Supplementary Table S1. Percentage ECD loss over time.

## Abbreviations

The following abbreviations are used in this manuscript:

FLACS	Femtosecond laser-assisted cataract surgery
CCS	Conventional cataract surgery
CCT	Central corneal thickness
CD	Endothelial cell density
BCVA	Best-corrected visual acuity
PT	Phacoemulsification time
IT	Intraoperative time
EPT	Effective phacoemulsification time
LOCSIII	Lens Opacities Classification System III
CV	Coefficient of variation of endothelial cell area
6A	Percentage of hexagonal cells
UT	Ultrasound time
