# Supplementary material for: Effect of Femtosecond Laser-Assisted Versus Conventional Clear Corneal Incisions on Endothelial Cell Density and Surgical Efficiency in Cataract Surgery
Source: J Clin Med. 2026 Jan 13;15(2):626. doi: 10.3390/jcm15020626 (PMC12841908; doi:10.3390/jcm15020626)
Supplement: Supplementary file 1 [file jcm-15-00626-s001.zip › jcm-3996502-supplementary.pdf]

Supplementary Table S1. Percentage ECD loss over time.

Time point	FLACS % loss	CCS % loss
Preop	0	0
Day 1	4.33	4.44
Day 12	4.37	5.06
Week4	5.47	7.09
Week6	8.86	7.39
Parameter Pair	Pearson r	*p*-value
CECD change vs EPT	−0.21	0.08
CECD change vs UT	−0.18	0.11
